# microRNA‐139‐5p confers sensitivity to antiepileptic drugs in refractory epilepsy by inhibition of MRP1

**DOI:** 10.1111/cns.13268

**Published:** 2019-11-21

**Authors:** Li Wang, Lifang Song, Xiaoyi Chen, Junfang Suo, Yanli Ma, Jinghe Shi, Kai Liu, Guohong Chen

**Affiliations:** ^1^ Department of Neurology Zhengzhou University Affiliated Children's Hospital (Zhengzhou Children's Hospital) Zhengzhou China

**Keywords:** drug resistance, microRNA‐139‐5p, multidrug resistance‐associated protein 1, neurons, refractory epilepsy

## Abstract

**Aim:**

Drug resistance is an intractable issue urgently needed to be overcome for improving efficiency of antiepileptic drugs in treating refractory epilepsy. microRNAs (miRNAs) have been proved as key regulators and therapeutic targets in epilepsy. Accordingly, the aim of the present study was to identify a novel differentially expressed miRNA which could improve the efficiency of antiepileptic drugs during the treatment of refractory epilepsy.

**Methods and Results:**

Serum samples were collected from children with refractory epilepsy. An in vivo refractory epilepsy model was developed in SD rats by electrical amygdala kindling. We identified that miR‐139‐5p was decreased and multidrug resistance‐associated protein 1 (MRP1) was remarkably upregulated in the serum samples from children with refractory epilepsy and the brain tissues from rat models of refractory epilepsy. After phenobarbitone injection in rat models of refractory epilepsy, the after discharging threshold in kindled amygdala was detected to screen out drug‐resistant rats. Dual‐luciferase reporter gene assay demonstrated that MRP1 was a target of miR‐139‐5p. In order to evaluate the effect of miR‐139‐5p/MRP1 axis on drug resistance of refractory epilepsy, we transfected plasmids into the hippocampus of drug‐resistant rats to alter the expression of miR‐139‐5p and MRP1. TUNEL staining and Nissl staining showed that miR‐139‐5p overexpression or MRP1 downregulation could reduce the apoptosis and promote survival of neurons, accompanied by alleviated neuronal damage.

**Conclusion:**

Collectively, these results suggest an important role of miR‐139‐5p/MRP1 axis in reducing the resistance of refractory epilepsy to antiepileptic drugs.

## 1 INTRODUCTION

Epilepsy represents a commonly occurring and detrimental neurological disorder accompanied by unprovoked spontaneous and recurrent seizures.^1^, ^2^ Previously released data supported that epilepsy, as a critical cause of disability and mortality, approximately affects over 70 million individuals across the globe.^3^ Despite the advances achieved in the first line of treatment of antiepileptic drugs, around one‐third of patients suffering from epilepsy run the risk of seizures refractory to pharmacotherapy.^4^ The major goal of treatment for epilepsy is to diminish the frequency of seizures and sustain seizure freedom so as to improve quality of life.^5^ Previous studies have provided explanation for the refractory or drug‐resistant epilepsy, the seizures of which tolerated or could not be controlled by at least two antiepileptic drugs for a specific epilepsy form.^6^, ^7^, ^8^ Thus, an in‐depth understanding of the mechanisms of antiepileptic drug resistance underscores the potential to aid developing novel therapeutic options for patients suffering from refractory epilepsy.

microRNAs (miRNAs), representing a family of small noncoding RNAs, could mediate the expression of various proteins by diminishing mRNA translation and stability and have the capacity to act as key regulators and therapeutic targets in the diagnosis and treatment of epilepsy.^9^ Recent studies have provided increasing evidence for the roles of miRNAs in refractory epilepsy, especially for their potentials to act as promising biomarkers to maintain stable disease status and predicting diagnosis and prognosis in the clinical scenario.^10^, ^11^ There is a paucity of evidence indicating the tumor‐suppressive role of microRNA‐139‐5p (miR‐139‐5p) in various cancers, such as attenuating growth of hepatocellular carcinoma,^12^ and reducing the resistance of colorectal cancer cells to 5‐fluorouracil chemotherapy.^13^ Interestingly, the high expression of miR‐139‐5p has been identified in the study of Alsharafi et al, which also proposed the potential use of miR‐139‐5p as a therapeutic target for temporal lobe epilepsy.^14^ It is important to note that miRNAs control the gene expression by means of binding to complementary sites on genes.^15^ This present study identified that multidrug resistance‐associated protein 1 (MRP1) was a target gene of miR‐139‐5p. Interestingly, MRP1 has been documented to modulate the pathological changes and drug resistance in refractory epilepsy in a rat model of chronic refractory epilepsy.^16^ Therefore, this current study aims to delineate the effect of miR‐139‐5p on the drug resistance in refractory epilepsy, with the involvement of MRP1.

## 2 MATERIALS AND METHODS

### 2.1 Ethical statement

This study was approved by the Ethics Committee in Zhengzhou University Affiliated Children's Hospital (Zhengzhou Children's Hospital). Informed consent documentation was obtained from the parents or guardians at enrollment. All the animal experimental protocols followed the Guide for the Care and Use of Laboratory Animals.

### 2.2 Study subjects

From June 2016 to June 2017, 61 children diagnosed with epilepsy at the Department of Neurology in Zhengzhou University Affiliated Children's Hospital (Zhengzhou Children's Hospital) were selected for the present study, comprising of 26 cases of refractory epilepsy and 35 cases of newly diagnosed epilepsy (NDE). Guidelines of the International League Against Epilepsy were applied to the diagnosis of refractory epilepsy.^17^, ^18^ Briefly, children with refractory epilepsy suffered from epileptic seizures at least four times per month. More than 2 AEDs were adopted for over 2 years along with effective drug concentration in blood which were unable to control the disease to affect daily life. Besides, they neither underwent space‐occupying lesions or progressive diseases of the central nervous system nor had a personal or family history of nervous system diseases except epilepsy. On the other hand, children with NDE were those who had a history of epileptic seizures and electroencephalogram (EEG) manifestations of epileptic discharge without exposure to AEDs. Additionally, 12 children with traumatic brain injury and eight with cerebrovascular malformation who did not receive any drug treatment were included as control. For gene expression analysis, 2 mL peripheral venous blood samples from each individual enrolled were collected into ethylene diamine tetraacetic acid (EDTA)‐containing tubes. The samples were then examined by reverse transcription quantitative polymerase chain reaction (RT‐qPCR) and Western blot analysis to determine the expression of miR‐139‐5p and MRP1.

### 2.3 Animal model

Healthy male Sprague Dawley (SD) rats (weight: 180‐220 g) were provided by the experimental animal center of Henan province (Zhengzhou, Henan, China). A total of 201 rats were kept under controlled environmental conditions at 18‐24°C with 60% humidity in a 12‐hour light/dark cycle and given ad libitum access to food and water. Then, five rats were assigned as controls receiving no treatment, while the rest were employed in order to establish the refractory epilepsy models.

The rat model of refractory epilepsy was developed by electrical amygdala kindling based on previously described methods.^19^ In brief, under pentobarbital sodium anesthesia (40 mg/kg, i.p.), an EEG electrode of nichrome wires was stereotaxically implanted in the amygdala. Based on existing stereotaxic atlases of the SD rat brain,^20^, ^21^ the stereotaxic coordinates for the amygdala were determined as follows: 3.0 mm posterior to bregma, 4.8 lateral to midline, and 8.8 below the dura mater. The stainless‐steel screws were then attached to the exposed skull for EEG recordings. With penicillin treatment (i.p.) in the first 3 days, the rats were allowed to recover for 7 days, followed by after discharge threshold (ADT) test. The parameters of stimulation were as follows: 16‐Hz frequency, biphasic square pulses of 1‐ms duration applied for 10 seconds. The intensity was delivered through a constant‐current stimulator producing square‐wave pulses and adjusted every 1 minute until a minimum AD of 3 seconds duration was evoked. Stimulation intensities began at 20 μA. If no AD was induced at this level, additional 20 μA intensity could be supplemented in the next trial until an AD was evoked. The lowest intensity of stimulation for producing an AD was designated as the ADT. The amygdala was stimulated with 400 μA intensity at 7‐minute intervals, and the rat was considered kindled when stage IV‐V seizures were observed. Seizure behavior was graded on the basis of Racine's classification (1972): stage I, facial clonus; stage II, head nodding; stage III, unilateral forelimb clonus; stage IV, bilateral forelimb clonus with rearing; stage V, generalized tonic‐clonic convulsions. In addition, 24 hours later after 10 consecutive stage V seizures were observed, the ADT of the amygdala‐kindled rat was detected.

### 2.4 Experimental animal

A total of 30 rats resistant to drugs were selected and classified into six groups: blank group (without treatment), miR‐139‐5p agomir group (injected with miR‐139‐5p agomir), agomir NC group (injected with agomir NC), sh‐MRP1 group (injected with lentiviral sh‐MRP1), (5) sh‐NC group (injected with lentiviral sh‐NC), and miR‐139‐5p agomir + oe‐MRP1 group (injected with miR‐139‐5p agomir and MRP1 overexpression vector). At the end of 24 hours post‐ADT test, the antiepileptic response to phenobarbitone (PB) was evaluated in the kindled rats by i.p. administration of 30 mg/kg PB. For control, normal saline was utilized instead of PB. The ADT was measured 1 hour later. Meanwhile, the blood from rat tail was extracted to determine drug concentration using a high‐performance liquid chromatography. In each rat, three trials with PB administration were performed at intervals of 1 week. Drug‐resistant rats and drug‐responsive rats were selected using the criteria proposed by Löscher et al using the ADT after saline injection as the control.^22^, ^23^ Drug‐resistant rats were defined as those not showing any increase or an average increase of no more than 20% in ADT upon repeated application of PB, while drug‐responsive rats showing at least 20% ADT increase in all three trials with PB. Rats showing variable responses were not included in the further experiments.

Next, the drug‐resistant rats were injected with a series of recombinant lentiviruses carrying miRNA agomir, gene‐specific short hairpin RNA (shRNA), or overexpression plasmids. Briefly, the selected fragments were cloned into the EcoRI and BamHI sites of pLVX‐IRES‐ZsGreenl vectors, which were then transformed into Escherichia coli DH5α cells. After amplification, the recombinant plasmids were extracted and identified by DNA sequencing. Subsequently, human embryonic kidney (HEK)‐293T cells were used to package the recombinant lentivirus vectors. Following 48‐72 hours incubation, cell supernatants containing the lentiviruses were harvested and then concentrated. The lentiviruses with a titer of 10^7^ TU/mL were subpacked and cryopreserved at −80°C for further use. After the drug‐resistant epileptic rats were induced, the rats were instantly anaesthetized with 1% pentobarbital sodium, shaved and injected with 4 μL lentiviruses at a speed of 0.5 μL/min into rat hippocampus. After 7 days of injection, the rats were euthanized.^24^, ^25^

### 2.5 Immunohistochemistry

Drug‐resistant, drug‐responsive, and control rats were euthanized to harvest brain tissues. Tissue samples were fixed in 10% formalin, paraffin‐embedded and then made into 4‐µm‐thick slices. Thereafter, the tissue slices were dried, dewaxed using xylene, and dehydrated using gradient ethanol. The slices were then blocked with 3% H_2_O_2_ (84885; Sigma‐Aldrich) at 37°C for 30 minutes. After phosphate‐buffered solution (PBS) washing for 3 minutes, the slices were boiled with 0.01 mol/L citric acid for 20 minutes at 95°C. Next, the slices were blocked with normal goat serum at 37°C for 1 hour and incubated with the primary antibody, mouse antibody to MRP1 (1:500, ab32574) at 4ºC overnight. On the following day, the secondary antibody, horseradish peroxidase (HRP)‐conjugated goat anti‐rabbit antibody (1:5000, ab205719; Abcam Inc) at room temperature for 30 minutes. Afterward, the slices were developed with diaminobenzidine (DAB), counterstained with hematoxylin and mounted. PBS, as a substitute for the primary antibody, was regarded as the negative control (NC). The slices were observed under a microscope, with five fields (400×) at high magnifications from each slice randomly selected in which 100 cells were counted. In the event that the percentage of positive cells in each field was more than 10%, the slice was then marked as positive.

### 2.6 Terminal deoxynucleotidyl transferase‐mediated dUTP nick‐end labeling (TUNEL) assay

After deparaffinization, the brain tissue samples were hydrated in gradient ethanol and treated with 3% H_2_O_2_ in methanol to block endogenous peroxidase. The cells growing on coverslips were fixed and then permeabilized with 0.1% Triton X‐100 (Sigma‐Aldrich) for 3 minutes. Following PBS washes, all samples were examined based on the instructions of a TUNEL detection kit (Roche). Following a 50‐minute incubation with 50 μL TdT labeling buffer containing TdT enzyme (1:9), the samples were reacted with 50 μL peroxidase at 37°C for 30 minutes, stained with DAB for 3 minutes, counterstained with hematoxylin for 3 seconds, and mounted with neutral balsam. Finally, five nonoverlapping views were randomly selected at high magnifications in a bid to calculate the TUNEL‐positive apoptotic cells. The rate of apoptosis was expressed as the percentage of apoptotic cells relative to the total number of cells.

### 2.7 Nissl staining

The brain tissue samples underwent a series of procedures for tissue slice preparations, including fixation, dehydration, and embedding. The slices were then placed on polylysine‐coated slides, dried, and hydrated in PBS for 10 minutes. After a 6‐8‐minute 0.1% cresyl violet staining at 37°C, the slices were rinsed with double‐distilled water for 3 minutes and decolorized in hydrochloric acid‐alcohol for 10 seconds, following by dehydration through conventional means. Subsequently, xylene was used to clear the slices and neutral balsam to seal. After slice drying, six nonoverlapping fields were randomly selected under a light microscope (40×; Leica) for neuron counting.

### 2.8 Dual‐luciferase reporter gene assay

The target gene of miR‐139‐5p were predicted by a biological software and further verified by the dual‐luciferase reporter gene assay. In brief, the putative miR‐139‐5p target binding sequences in MRP1 3’‐untranslated region (3’‐UTR) were designed and synthesized, followed by a site‐specific mutagenesis. The sequences containing the binding sites or mutagenesis were cloned into the pmiR‐RB‐REPORT^™^ luciferase reporter vector (Guangzhou RiboBio Co., Ltd.) which was pretreated with restriction enzymes. miR‐139‐5p mimic, mimic NC, miR‐139‐5p inhibitor, and inhibitor NC were, respectively, co‐transduced with the constructed luciferase reporter vectors into human embryonic kidney (HEK)‐293T cells. The luciferase activity was measured at the 48th h after transduction using the Luciferase Reporter Assay Kit (E1910; Promega). The relative luciferase activity was expressed as the ratio of firefly luciferase activity to the activity of internal reference renilla luciferase.

### 2.9 RNA extraction and quantification

Total RNA was extracted using the Trizol kit (15596026; Invitrogen). The integrity, concentration, and purity of RNA were tested with electrophoresis on 1% agarose gel and a NanoDrop ND‐1000 spectrophotometer (Thermo Fisher Scientific). Total RNA was reversely transcribed into complementary DNA (cDNA) with the PrimeScript RT reagent kit (RR047A; Takara Bio Inc) in a 20‐μL reaction system. The cDNA of miRNA was synthesized with the Mir‐X miRNA First Strand Synthesis Kit (638315; Clontech) following manufacturer's recommendations. The quantitative polymerase chain reaction (qPCR) was conducted with 20 μL of reaction system using SYBR^®^ Premix Ex TaqTM II (TaKaRa Biotechnology Co., Ltd.) on an ABI7500 instrument (Applied Biosystems, Inc). The primers used are listed in Table 1, which were synthesized by Shanghai Sangon Biotechnology Co., Ltd. Glyceraldehyde‐3‐phosphate dehydrogenase (GAPDH) or U6 was used as the internal references, and the fold changes were calculated by means of relative quantification (2^−ΔΔCt^ method).^26^ Each experiment was conducted with at least three biological repeats.

**Table 1:** Primer sequences for RT‐qPCR

Gene	Forward primer (5′‐3′)	Reverse primer (5′‐3′)
Rattus norvegicus miR‐139‐5p	ACACTCCAGCTGGGTCTACAGTGCAC	TGGTGTCGTGGAGTCG
Rattus norvegicus MRP1	CCATTCAGGCCGGTAGAGT	TCATGGTTCAGCTTGTCAGG
Rattus norvegicus U6	GCTTCGGCAGCACATATACTAAAAT	CGCTTCACGAATTTGCGTGCAT
Rattus norvegicus GAPDH	CATCAACGACCCCTTCATTG	GAAGATGGTGATGGGTTTCC
Homo sapiens miR‐139‐5p	GCCTCTACAGTGCACGTGTCTC	CGCTGTTCTCATCTGTCTCGC
Homo sapiens MRP1	TTCCGGAACTACTGCCTGCGCTA	GGGTCCTGGGGGATGATGGTGA
Homo sapiens U6	GCTTCGGCAGCAGCACATATACTAAAAT	CGCTTCACGAATTTGAGTGTCAT
Homo sapiens GADPH	CGCTTCGGCAGCACATATACTA	CGCTTCACGAATTTGCGTGTCA

### 2.10 Western blot analysis

Total proteins were extracted using radio‐immunoprecipitation assay (RIPA) lysis buffer (P0013B; Beyotime Biotechnology Co., Ltd.) containing phenylmethylsulfonyl and phosphatase inhibitors. After a 30‐min incubation on ice, the samples were centrifuged at 12 000 rpm for 10 minutes at 4°C, followed by the supernatant collection. A bicinchoninic acid (BCA) kit (Beyotime Biotechnology Co., Ltd.) was utilized to determine protein concentration. Next, 30 µg protein was separated by sodium dodecyl sulfonate‐polyacrylamide gel electrophoresis and then transferred onto a nitrocellulose membrane using wet transfer apparatus. After that, the membrane was blocked with 5% skimmed milk in Tris‐buffered saline Tween (TBST) buffer for 1.5 hours and incubated with the primary antibody to MRP1 (1:500; ab32574, Abcam) at 4°C overnight. On the following day, the membrane was further incubated with the secondary antibody, horseradish peroxidase (HRP)‐conjugated goat anti‐rabbit antibody to immunoglobulin G (IgG; 1:2000‐50000; ab205718) for 2 hours at room temperature. Subsequently, the protein bands were visualized with an enhanced chemiluminescence (ECL) reagent, which were imaged on SmartView Pro 2000 (UVCI‐2100; Major Science). Image analysis was followed using Quantity One software. The target proteins were quantified as relative gray values against the internal reference GAPDH.

### 2.11 Statistical analysis

All experimental data were analyzed using SPSS 21.0 statistical software (IBM Corp.). Measurement data are presented as mean ± standard deviation. Differences between two groups were compared using unpaired *t* test. Comparisons among multiple groups were analyzed using one‐way analysis of variance (ANOVA) with Tukey's post hoc test. A *P* value < .05 was considered statistically significant.

## 3 RESULTS

### 3.1 miR‐139‐5p is decreased while MRP1 is increased in serum of children with refractory epilepsy

**Figure 1:**
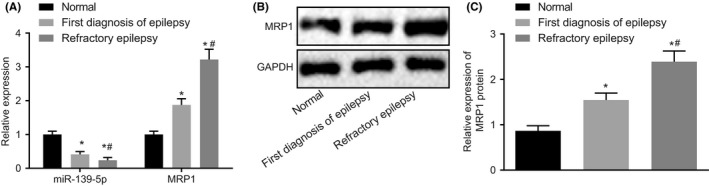
Circulating miR‐139‐5p is downregulated while MRP1 is robustly induced in children with epilepsy. A, Relative expression of miR‐139‐5p and MRP1 mRNA in serum (U6 or GAPDH as the internal reference) evaluated by RT‐qPCR; B, the protein bands of MRP1 in serum measured by Western blot analysis (GAPDH as the internal reference); C, quantitative analysis of MRP1 protein expression (GAPDH as the internal reference); *P < .05 vs the serum from normal children; #*P* < .05 vs the serum from NDE children. Measurement data were expressed as mean ± standard deviation. The comparisons among multiple groups were analyzed by one‐way ANOVA with Tukey's post hoc test. 20 normal children, 35 NDE children, and 26 children with refractory epilepsy

Initially, we performed RT‐qPCR to examine miR‐139‐5p and MRP1 mRNA expression in serum samples extracted from 20 normal children, 35 NDE children, and 26 children with refractory epilepsy. As shown in Figure 1A, the expression of miR‐139‐5p was decreased while MRP1 mRNA was increased in serum of NDE children compared with the serum from normal children (*P* < .05). Besides, the serum from children with refractory epilepsy showed significantly lower miR‐139‐5p expression and higher mRNA expression of MRP1 compared with the serum from NDE children. Western blot analysis was used to detect MRP1 protein expression in serum samples, Figure 1B,C showed that the protein expression of MRP1 was lower in the serum of normal children than that of serum in NDE children, while MRP1 was increased in serum of refractory epilepsy than that of serum in NDE children (*P* < .05). The aforementioned results suggest that the expression of miR‐139‐5p in the serum from children with refractory epilepsy is at lowest levels, accompanied with robust expression of MRP1.

### 3.2 miR‐139‐5p is downregulated while MRP1 is robustly induced in brain tissues of the rat model of refractory epilepsy

**Figure 2:**
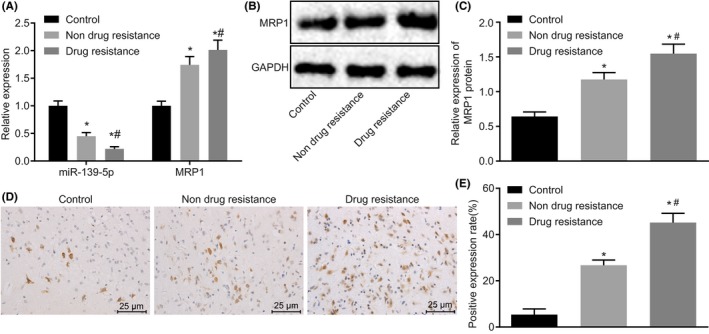
miR‐139‐5p is decreased while MRP1 is upregulated in brain tissues of rats with refractory epilepsy. A, relative miR‐139‐5p expression and MRP1 mRNA expression in brain tissues evaluated by RT‐qPCR (U6 or GAPDH as the internal reference); B, the protein bands of MRP1 in brain tissues determined by Western blot analysis (GAPDH as the internal reference); C, quantitative analysis of MRP1 protein expression in brain tissues (GAPDH as the internal reference); D, immunohistochemical staining of MRP1 in brain tissues (400×); E, positive expression rate of MRP1 protein in brain tissues determined by immunohistochemistry; *P < .05 vs the control rats; #*P* < .05 vs the drug‐responsive rats. Measurement data were expressed as mean ± standard deviation. The comparisons among multiple groups were analyzed by one‐way ANOVA with Tukey's post hoc test. Control group n = 5; Drug‐resistant n = 10; Drug‐responsive n = 57

Subsequently, we developed the refractory epilepsy model in 196 SD rats by electrical amygdala kindling. The results confirmed that 170 rats were successfully kindled, indicating a kindling acquisition rate of 86.73%. Next, 165 kindled rats were injected with PB for identification of drug resistance. For control group, five kindled rats were treated with normal saline instead of PB. PB responders and nonresponders after kindling acquisition were selected according to ADT changes. Compared with the control rats, 45 rats showed no increase or an average increase of no more than 20% in ADT upon repeated application of PB which were drug‐resistant (the resistance rate was 27.27%); and 57 rats presented at least 20% ADT increase in all three trials with PB that were drug‐responsive (the response rate was 34.55%). Besides, 30 rats revealed variable responses and the variation rate was 18.18%. The values of ADT of drug‐resistant and drug‐responsive rats before/after kindling acquisition or drug administration were displayed in Table 2. The drug‐resistant rats showed no marked changes among these ADT values (*P* > .05). Compared with the ADT before drug administration, the ADT of drug‐responsive rats was remarkably elevated after drug administration (*P* < .05). Next, RT‐qPCR and Western blot analysis were conducted to determine the expression of miR‐139‐5p and MRP1 in the brain tissues from kindled rats after drug administration. miR‐139‐5p was decreased, while MRP1 mRNA and protein expression were increased in the drug‐resistant and drug‐responsive rats in comparison with the control rats (*P* < .05; Figure 2A‐C). Immunohistochemistry results (Figure 2D,E) further revealed that the drug‐resistant and drug‐responsive rats had higher positive rate of MRP1 protein and the drug‐resistant rats showed more obvious increase compared with the control rats (*P* < .05). Collectively, these results verified that miR‐139‐5p is decreased while MRP1 is increased in the brain tissues from rat models of refractory epilepsy.

**Table 2:** The values of ADT of drug‐resistant and drug‐responsive rats before/after kindling acquisition or drug administration

Treatment	Drug‐resistant (n = 10)	Drug‐responsive (n = 57)
ADT before kindling acquisition	153 ± 12.6	140 ± 14.3
ADT after kindling acquisition	142 ± 8.47	135 ± 13.6
ADT before drug administration	145 ± 8.65	138 ± 13.5
ADT after drug administration	150 ± 6.94	246 ± 63.3*

Note

Measurement data were expressed as mean ± standard deviation. The comparisons among multiple groups were analyzed by one‐way ANOVA with Tukey's post hoc test.

* *P* < .05 vs values before kindling acquisition or drug administration.

### 3.3 miR‐139‐5p targets and negatively regulates MRP1 expression

**Figure 3:**
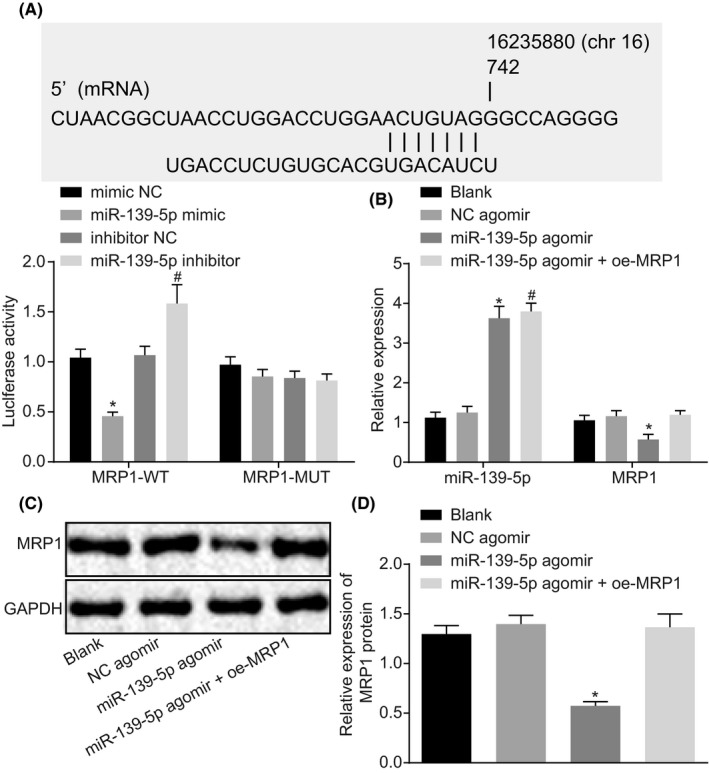
MRP1 is a target of miR‐139‐5p. A, putative binding sites between miR‐139‐5p and MRP1 3’‐UTR and the luciferase activities; the rats were treated with miR‐139‐5p agomir alone or in the presence of oe‐MRP1; B, relative miR‐139‐5p expression and MRP1 mRNA expression in brain tissues evaluated by RT‐qPCR (U6 or GAPDH as the internal reference). n = 5; C, the protein bands of MRP1 in brain tissues determined by Western blot analysis (GAPDH as the internal reference); D, quantitative analysis of MRP1 protein expression in brain tissues (GAPDH as the internal reference). n = 5; *P < .05 vs the blank group; #*P* < .05 vs the NC agomir group. Measurement data were expressed as mean ± standard error. The comparison between two groups was analyzed by unpaired *t* test and the comparisons among multiple groups by one‐way ANOVA with Tukey's post hoc test. Each experiment was repeated three times

Bioinformatics analysis further predicted that MRP1 might be a target gene of miR‐139‐5p. In order to determine whether miR‐139‐5p targeted MRP1, the dual‐luciferase reporter gene assay was performed. The results showed no significant differences in the luciferase activity in HEK‐293T cells treated with miR‐139‐5p mimic and a vector containing the MRP1 3’‐UTR with recognizing sites mutated. Nevertheless, the luciferase activity was markedly reduced in HEK‐293T cells treated with miR‐139‐5p mimic and MRP1‐wild type (WT) vs cotreatment of mimic NC and MRP1‐WT. Conversely, the cotransfection of miR‐139‐5p inhibitor and MRP1‐WT resulted in enhanced luciferase activity in HEK‐293T cells relative to inhibitor NC and MRP1‐WT (Figure 3A,B). Therefore, miR‐139‐5p targeted MRP1 and negatively regulated its expression. Moreover, we evaluated the interaction of miR‐139‐5p and MRP1 in the brain tissues of rats with refractory epilepsy. As displayed in Figure 3C,D, the expression of MRP1 in the brain tissues was decreased and miR‐139‐5p was restored after miR‐139‐5p agomir transfection. These results demonstrated that MRP1 is a target gene of miR‐139‐5p.

### 3.4 miR‐139‐5p enhances drug sensitivity of refractory epilepsy by downregulating MRP1

**Figure 4:**
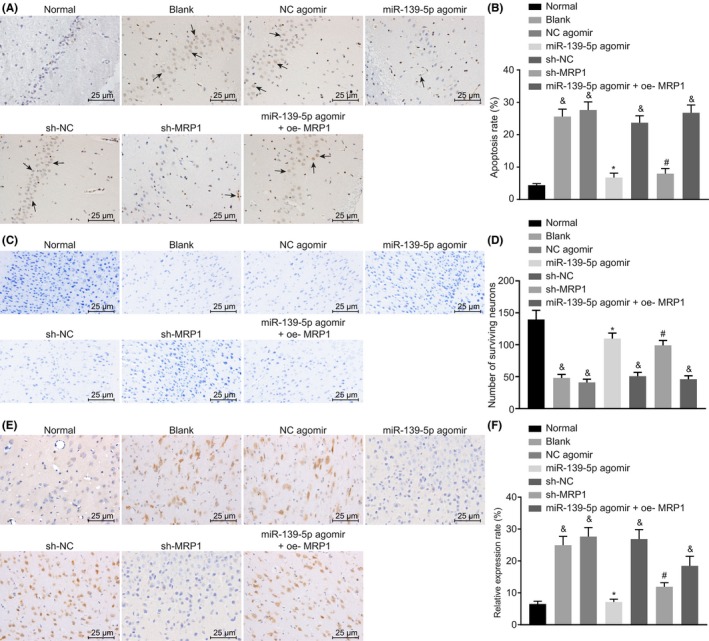
miR‐139‐5p reduces drug resistance of refractory epilepsy *via* downregulating MRP1. The rats were treated with sh‐MRP1, miR‐139‐5p agomir alone or in the presence of oe‐MRP1. A, TUNEL staining of brain tissues of rats where arrows indicate TUNEL‐positive cells (400×); B, statistical analysis of apoptosis rate in brain tissues of rats detected by TUNEL staining; C, Nissl staining of brain tissues of rats (400×); D, number of surviving neurons in brain tissues of rats detected by Nissl staining; E, immunohistochemical staining of MRP1 in brain tissues of rats (400×); F, relative positive rate of MRP1 in brain tissues of rats; and *P* < .05 vs the normal group; *P < .05 vs the NC agomir group; #*P* < .05 vs the sh‐NC agomir group. Measurement data were expressed as mean ± standard deviation. Differences between two groups were compared using unpaired *t* test. The comparisons among multiple groups were analyzed by one‐way ANOVA with Tukey's post hoc test. n = 5

In order to evaluate the role of miR‐139‐5p/MRP1 axis in drug‐resistant refractory epilepsy, we delivered a series of plasmids to upregulate or downregulate miR‐139‐5p and MRP1 in drug‐resistant rats with refractory epilepsy. The results of TUNEL assay in Figure 4A,B showed that compared with normal rats, TUNEL‐positive cells were increased significantly in rats with refractory epilepsy injected with NC agomir, sh‐NC, and miR‐139‐5p agomir + oe‐MRP1. Besides, apoptotic cell number was reduced in the brain tissues induced by overexpression of miR‐139‐5p or downregulation of MRP1. Nissl staining was performed to observe neuronal damage. As shown in Figure 4C,D, the upregulation of miR‐139‐5p and overexpression of MRP1 together could trigger significant damage in hippocampal neurons: disordered cell arrangement, incomplete cell structure, cytoplasmic condensation, karyopyknosis, and reduction of Nissl bodies in cytoplasm. Importantly, the above neuronal damage could be markedly ameliorated in the event of miR‐139‐5p upregulation or MRP1 inhibition, as evidenced by a large number of evenly aligned dense vertebral body with clear structure, uniform staining distribution, and rich Nissl corpuscles in cytoplasm. In comparison to the normal rats, rats with refractory epilepsy injected with NC agomir, sh‐NC, and miR‐139‐5p agomir + oe‐MRP1 displayed notably reduced surviving neurons, whereas overexpression of miR‐139‐5p or downregulation of MRP1 contributed to enhanced surviving neurons. The expression of MRP1 rat tissues was detected by immunohistochemistry (Figure 4E,F). The results illustrated that the MRP1 positive cells in rats with refractory epilepsy injected with NC agomir, sh‐NC and miR‐139‐5p agomir + oe‐MRP1 were significantly higher than those in normal rats. Consistently, overexpression of miR‐139‐5p or downregulation of MRP1 led to a decline in MRP1 positive cells. Moreover, there was no significant difference in ADT before/after kindling acquisition and ADT before/after drug administration among rats with refractory epilepsy injected with NC agomir, sh‐NC and miR‐139‐5p agomir + oe‐MRP1; while the ADT after drug administration in the rats with overexpression of miR‐139‐5p or downregulation of MRP1 was significantly higher than that before the administration (Table 3). As a consequence, miR‐139‐5p restoration or MRP1 depletion could reduce drug resistance of refractory epilepsy.

**Table 3:** The values of ADT before/after kindling acquisition or drug administration of rats with refractory epilepsy treated with miR‐139‐5p restoration or MRP1 depletion

Group	ADT before kindling acquisition	ADT after kindling acquisition	ADT before drug administration	ADT after drug administration
Blank	154 ± 12.6	142 ± 10.7	143 ± 8.98	153 ± 10.7
NC agomir	155 ± 10.4	142 ± 8.46	142 ± 8.86	151 ± 9.94
miR‐139‐5p agmir	143 ± 9.65	133 ± 9.05	143 ± 9.65	240 ± 21.6^**^
sh‐NC	152 ± 11.0	145 ± 9.64	145 ± 10.6	152 ± 11.5
sh‐MRP1	140 ± 9.31	131 ± 8.31	139 ± 8.13	238 ± 24.6^**^
miR‐139‐5p agmir + oe‐MRP1	156 ± 15.6	146 ± 12.6	141 ± 9.61	149 ± 10.6

Note

Measurement data were expressed as mean ± standard deviation. The comparisons among multiple groups were analyzed by one‐way ANOVA with Tukey's post hoc test. n = 10.

**P < .05 vs values before kindling acquisition or drug administration.

## 4 DISCUSSION

Epilepsy is accompanied by frequently recurrent seizures, which usually leads to debilitated quality of life.^27^ Interestingly, the pathogenesis of epilepsy has been verified to be associated with genes that modulate inflammation, cell death, neural signaling, and synaptic structure.^28^ In addition, a hypothesis has been previously demonstrated that the aberrant alterations in gene expression results in aberrant hyperexcitability, which may cause repeated seizures.^1^ It is significant to note the epigenetic mechanisms previously delineated to characterize the development of drug resistance in epilepsy, during which the altered gene expression has a role to play during antiepileptic drug uptake.^29^, ^30^ Here, our study highlighted the involvement of miR‐139‐5p in the translational suppression of MRP1, resulting in the inhibition of drug resistance in refractory epilepsy.

An increasing number of studies have documented the potential roles of multiple miRNAs in epilepsy, which provided better understanding of the pathogenesis, treatment, and diagnosis.^31^, ^32^ The current antiepileptic drugs have been proved to generate effects on the expression of miRNAs, and the characterization of these effects on the pharmacoresistance and behavioral alterations is clinically valuable.^33^ Additionally, abnormal changes in miRNA expression in the hippocampus of temporal lobe epilepsy and in neural tissues from animal models of epilepsy is clearly associated with neurotransmitter signaling, neuroinflammation, as well as transcription factors.^9^ Neuroprotective effects of miR‐344a have been delineated in seizures, whereby an improvement in seizure behavior and a decrease in neurological impairments in the cortex in response to the treatment of miR‐334a agomir.^34^ It is worth noting that Alsharafi and his colleagues have proved that downregulated miR‐139‐5p in hippocampal tissues modulates the NR2A‐containing NMDA receptor during different phases of status epilepticus.^14^ However, they failed to state clearly the cell activities of neurons and the effect of miR‐139‐5p on the pharmacoresistance, which limited the clinical values. In the present study, the key observations and findings indicated that miR‐139‐5p was decreased in the serum samples of children with refractory epilepsy and the brain tissues from rats of refractory epilepsy.

Furthermore, we also revealed that upregulation of miR‐139‐5p reduced the apoptosis and promote survival of neurons, which alleviated neuronal damage. It has been well established that miRNAs could be used as therapeutic biomarkers due to their differential levels in epilepsy, in addition to availability in biofluids.^35^, ^36^ Moreover, the participation of neuronal death in chronic epilepsy and cognitive decline has been previously reported.^37^ The further delineation of the regulatory mechanisms of miR‐139‐5p underlying refractory epilepsy is valuable, there is a great potential to translate the findings to clinical applications for patients with refractory epilepsy. Based on the findings of the prior study, the expression of miR‐139‐5p shared association with the cortical development, and the upregulation of miR‐139‐5p could attenuate the damaged cortex, which may be due to the regulatory effect of miR‐139‐5p on the migration of neurons in the cortex by targeting Lis1.^38^ Moreover, miR‐139‐5p agomir attenuates brain damage via inhibition of human growth transformation‐dependent protein.^39^ Thus, the upregulation of miR‐139‐5p has the great potential to prevent further epilepsy progression and to lessen antiepileptic drug resistance.

Notably, MRP1 was confirmed as a target of miR‐139‐5p. Downregulated MRP1 was found to reduce the apoptosis and promote survival of neurons, accompanied by alleviated neuronal damage. Evidence has been provided by a previous study that miR‐139‐5p negatively regulated the expression of the downstream molecule MRP1, thereby diminishing the chemoresistance of colorectal cancer cells.^40^ MRPs have been reported to possess the ability to transport amphiphilic organic anions such as drugs conjugated to sulfate, glutathione, and phosphate, as well as neutral organic drugs.^41^ Accordingly, it has been hypothesized that MRPs have the capacity to transport the antiepileptic drugs as well as corresponding metabolites and control the access to the brain.^42^ In addition, the expression of MRP1 has been observed in choroid plexus epithelial cells at the basolateral membrane, and poorly expressed MRP1 at the luminal membrane of endothelial cells has been found at the blood‐brain barrier.^43^ Besides, highly expressed MRP1 has been verified in astrocytes and blood vessels of the brain following acute status epilepticus and during chronic epilepsy.^44^ Therefore, the role of miR‐139‐5p in augmenting sensitivity to antiepileptic drugs was achieved by suppressing expression of MRP1.

The key findings of the present study provide evidence emphasizing the inhibitory effect of miR‐139‐5p on resistance to antiepileptic drugs, which also conferred neuroprotection against neuronal apoptosis by targeting MRP1 in refractory epilepsy. However, our study did not focus on the expression of other target proteins. In addition, the limited studies have investigated the role of miR‐139‐5p in antiepileptic drug resistance in refractory epilepsy, which may lead to insufficient data and reference materials. Future investigations are required to extend pharmacoresistance into other factors.

## CONFLICT OF INTEREST

We declare that we have no conflicts of interest.
