# Corrigendum

**DOI:** 10.1111/cns.13884

**Published:** 2022-06-17

In Wang et al.,^1^ the authors noticed that the same figure was accidently presented in Figure 4c and e.

The corrected Figure 4 is given below:

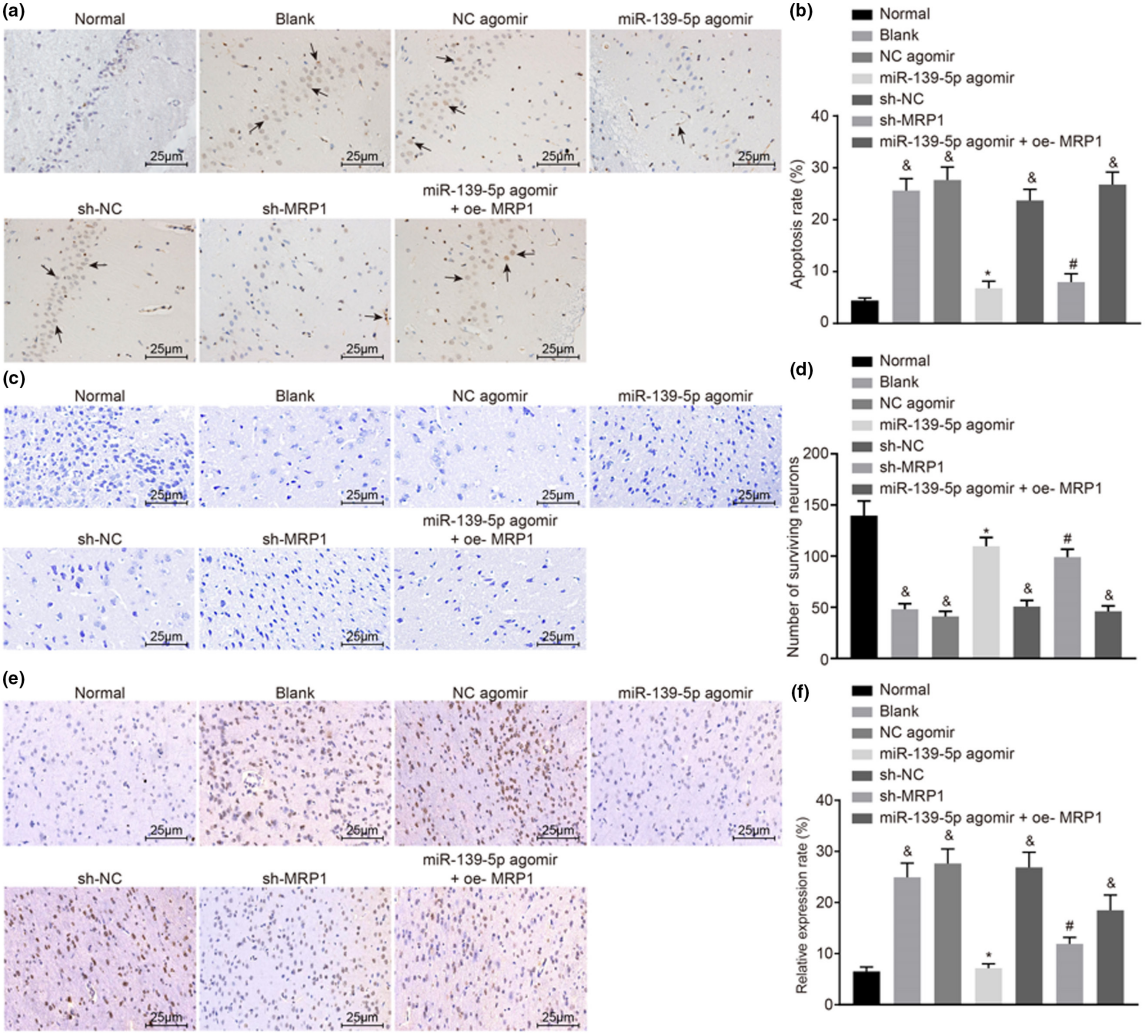

The authors apologize for this error.
